# Interfacial Interaction in NiFe LDH/NiS_2_/VS_2_ for Enhanced Electrocatalytic Water Splitting

**DOI:** 10.3390/molecules29050951

**Published:** 2024-02-21

**Authors:** Tingxia Wang, Xu Zhang, Xiaojiao Yu, Junpeng Li, Kai Wang, Jinfen Niu

**Affiliations:** School of Science, Xi’an University of Technology, Xi’an 710054, China; xuzhang0501@126.com (X.Z.); junpengljp@163.com (J.L.); kw225713@163.com (K.W.); niujinfen@xaut.edu.cn (J.N.)

**Keywords:** vanadium disulfide, nickel disulfide, layered double hydroxide, interfacial regulation, electrocatalytic overall water splitting, hydrogen/oxygen evolution reaction

## Abstract

A bifunctional electrocatalyst with high efficiency and low costs for overall water splitting is critical to achieving a green hydrogen economy and coping with the energy crisis. However, developing robust electrocatalysts still faces huge challenges, owing to unsatisfactory electron transfer and inherent activity. Herein, NiFe LDH/NiS_2_/VS_2_ heterojunctions have been designed as freestanding bifunctional electrocatalysts to split water, exhibiting enhanced electron transfer and abundant catalytic sites. The optimum NiFe LDH/NiS_2_/VS_2_ electrocatalyst exhibits a small overpotential of 380 mV at 10 mA cm^−2^ for overall water splitting and superior electrocatalytic performance in both hydrogen and oxygen evolution reactions (HER/OER). Specifically, the electrocatalyst requires overpotentials of 76 and 286 mV at 10 mA cm^−2^ for HER and OER, respectively, in alkaline electrolytes, which originate from the synergistic interaction among the facilitated electron transfer and increasingly exposed active sites due to the modulation of interfaces and construction of heterojunctions.

## 1. Introduction

Electrocatalytic overall water splitting, as a promising green hydrogen (ideal zero-carbon energy carrier)-producing strategy, has attracted more attention for developing carbon-neutral alternatives [1,2,3]. However, electrocatalytic water-splitting activity is severely limited, owing to the sluggish catalytic kinetics for OER and HER [4,5,6,7]. Electrocatalysts of excellent efficiency are essential to reducing the overpotential by lowering the activation energy of water splitting [8,9]. At present, although IrO_2_, RuO_2_ and Pt-based materials have been employed as highly effective electrocatalysts for OER and HER, respectively, their scarcity and high price have hampered their industrialized application on a large scale [10,11]. The preparation of cost-effective bifunctional electrocatalysts with superior activity for OER and HER is crucial to promoting the practical applications and industrialized development of overall electrocatalytic water splitting.

Non-noble transition metal sulfides (TMSs) have presented themselves as appealing catalysts for both HER and OER on the basis of their favorable electrocatalytic activity and electrical conductivity [12,13]. Recently, vanadium disulfide (VS_2_) has attracted considerable attention as a candidate electrocatalyst, owing to its intrinsic catalytic properties, which exhibit weak van der Waals interlayer interactions and an interlayer spacing of 5.76 Å [14]. In particular, benefiting from its metallic conductivity and electrocatalytic active sites both on the surface and at the active edge, the 1T phase VS_2_ has been widely employed as an electrocatalyst for HER and OER [15]. Nevertheless, previous research reveals that individual TMSs exhibit insufficient active sites due to their strong van der Waals forces [16]. Massive efforts have been directed at constructing multi-component nanomaterial, which has been expected to improve its electrocatalytic activity by providing an abundance of active sites [17,18]. Ru cluster-modified VS_2_ can achieve a current density of 50 mA cm^−2^ at an overpotential of 245 mV, and this can be attributed to the optimization of intermediate adsorption/desorption through the interactions between Ru and VS_2_ and the sufficient catalytic sites afforded by electro-oxidized of Ru species [19]. Benefiting from induced-layer electron transfer between NiCo_2_S_4_ and ReS_2_ interfaces, the NiCo_2_S_4_/ReS_2_ nanocomposite showed excellent HER properties in both acidic and alkaline conditions [20]. The CoS_2_-ReS_2_ heterojunction exhibited improved electrocatalytic activity for HER and OER, which can be accounted for by the rich catalytic sites on the edge and the optimized catalytic kinetics of the interfacial modulation of ReS_2_ via modifying CoS_2_ [21]. Therefore, constructing binary transition metal sulfide-based electrocatalysts is beneficial to creating abundant catalytic active sites.

Interfacial regulation has been regarded as a powerful method to adjust electronic structure and catalytic kinetics to significantly enhance electrocatalysis [22,23,24,25,26]. NiFe layered double hydroxides (LDHs) have been widely investigated for OER because they can convert into γ-NiOOH by surface reconstruction, which has been proven to be the actual active sites [27,28]. Nevertheless, NiFe LDH exhibits limited electrocatalytic activity for OER due to weak conductivity and insufficient active sites [29]. Constructing heterostructures has been employed to improve its intrinsic catalytic activity by facilitating electron transfer kinetics and promoting the conversion of NiFe LDH into γ-NiOOH active species [30]. Urchin-like Co_9_S_8_@NiFe layered double-hydroxide heterostructured hollow spheres have been designed to enhance the electrocatalytic OER process by improving electrical conductivity and reducing the reaction energy barrier, requiring a smaller overpotential of 220 mV at 10 mA cm^−2^ [31]. By virtue of the abundant Ni_3_S_2_-NiFe LDH interfaces, this catalyst is regarded as one of the most productive non-precious metal-based OER catalysts [32]. Furthermore, the in-situ growth of heterogeneous structure electrocatalysts using a binder-free method can provide intimate contact between multi-components and electrodes, which can induce facilitated electron transfer [33,34]. Electrodeposition is a useful technique to rapidly fabricate multi-component nanomaterials through an in-situ growth process [35,36].

Inspired by the above-mentioned facts, a heterogeneous structure-based binary metal sulfide-modified carbon cloth (NiS_2_/VS_2_) has been constructed via a one-step hydrothermal process. Subsequently, an active OER electrocatalyst, NiFe LDH, was integrated into the surface of NiS_2_/VS_2_ (NiFe LDH/NiS_2_/VS_2_) using a facile electrochemical deposition method, which was simultaneously exploited as a bifunctional electrocatalyst for both HER and OER. This designed heterostructure of NiFe LDH/NiS_2_/VS_2_ nanosheets exhibits interfacial charge transfer from NiS_2_ to VS_2_ and promotes electron transfer during the electrocatalytic process. Moreover, the formed porous nanoflower-like structure is favorable for exposing more active sites and facilitating bubble releasing, all of which can promote the electrocatalytic performance for overall water splitting. As a bifunctional electrocatalyst, the obtained NiFe LDH/NiS_2_/VS_2_ can deliver a current density of 10 mA cm^−2^ at an overpotential of 76 and 286 mV severally for HER and OER, together with excellent electrocatalytic stability. Furthermore, the solar-driven electrocatalytic overall splitting system constructed by NiFe LDH/NiS_2_/VS_2_ demonstrated that it has good potential for practical applications.

## 2. Results and Discussion

### 2.1. Characterization of Samples

**Scheme 1:**
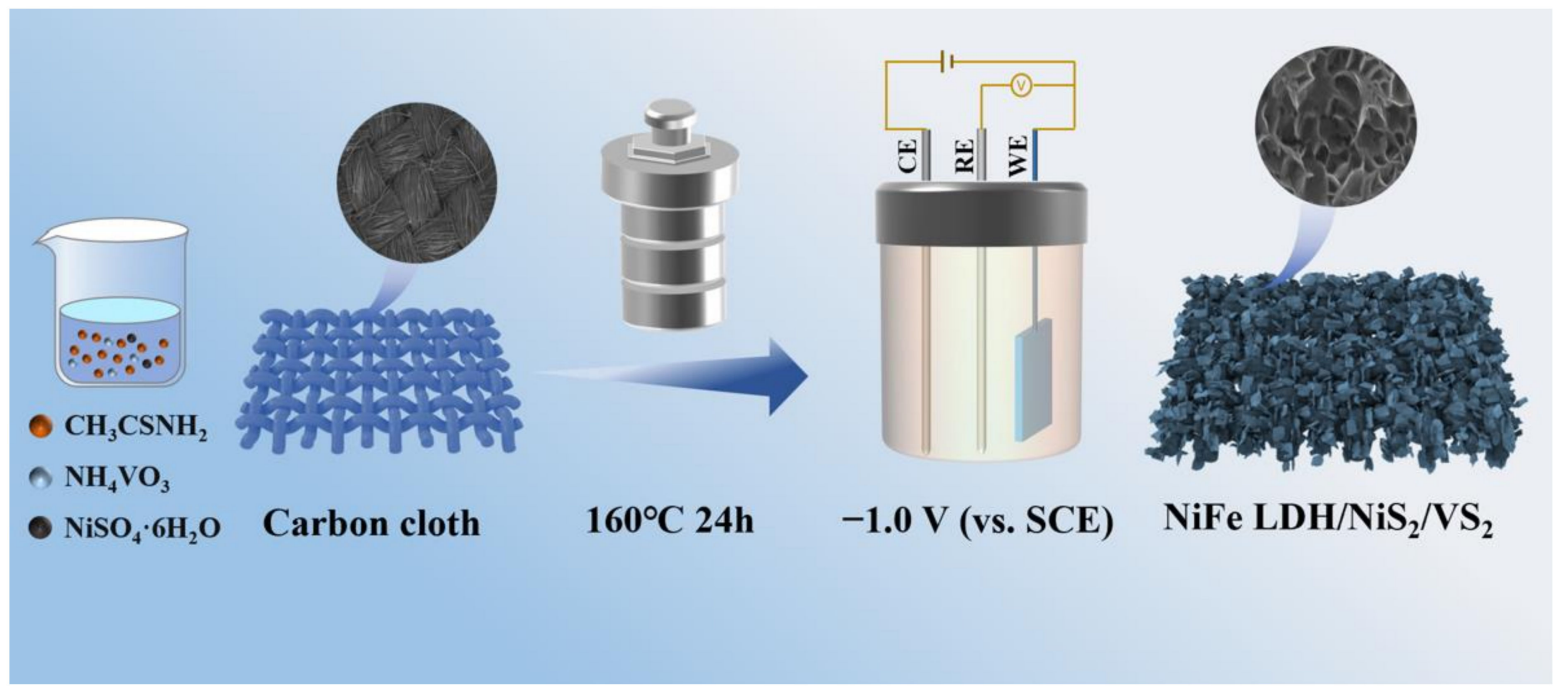
Schematic illustration of the preparation process of NiFe LDH/NiS_2_/VS_2_.

The synthetic pathway of NiFe LDH/NiS_2_/VS_2_ is schematically illustrated in Scheme 1, in which VS_2_ was in-situ deposited on the surface of carbon cloth (decorated as VS_2_/CC) by a facile hydrothermal method. Similarly, the NiS_2_/VS_2_ decorated CC was constructed by the above-mentioned method with the addition of NiSO_4_·6H_2_O. Subsequently, the NiFe LDH was modified onto the surface of NiS_2_/VS_2_ via a simple electrodeposition method, resulting in NiFe LDH/NiS_2_/VS_2_.

The phase and composition of pure VS_2_, NiS_2_/VS_2_ and NiFe LDH/NiS_2_/VS_2_ were measured by XRD. As shown in Figure S1a, the diffraction peaks located at 2θ values of 15.2, 35.64, 45.07, 47.06 and 57.02° can be ascribed to the (001), (011), (012), (003) and (110) lattice planes of VS_2_ (JCPDS No. 89-1640) [37,38]. The additional peaks at 31.58, 38.76, 53.43, 58.50 and 60.91° appeared when NiS_2_ was modified on the surface of VS_2_, which can be indexed to the (200), (211), (311), (230) and (321) plane of NiS_2_ (JCPDS No. 11-0099), respectively [39,40,41]. Compared with the XRD pattern of NiS_2_/VS_2_, the characteristic diffraction peaks at 11.26 and 35.28° appeared after the electrodeposition of NiFe LDH, which can be assigned to the (003) and (006) planes of NiFe LDH species (JCPDS No. 51-0463) [42,43]. The above-mentioned results suggest the co-existence of VS_2_, NiS_2_ and NiFe LDH in NiFe LDH/NiS_2_/VS_2_.

**Figure 1:**
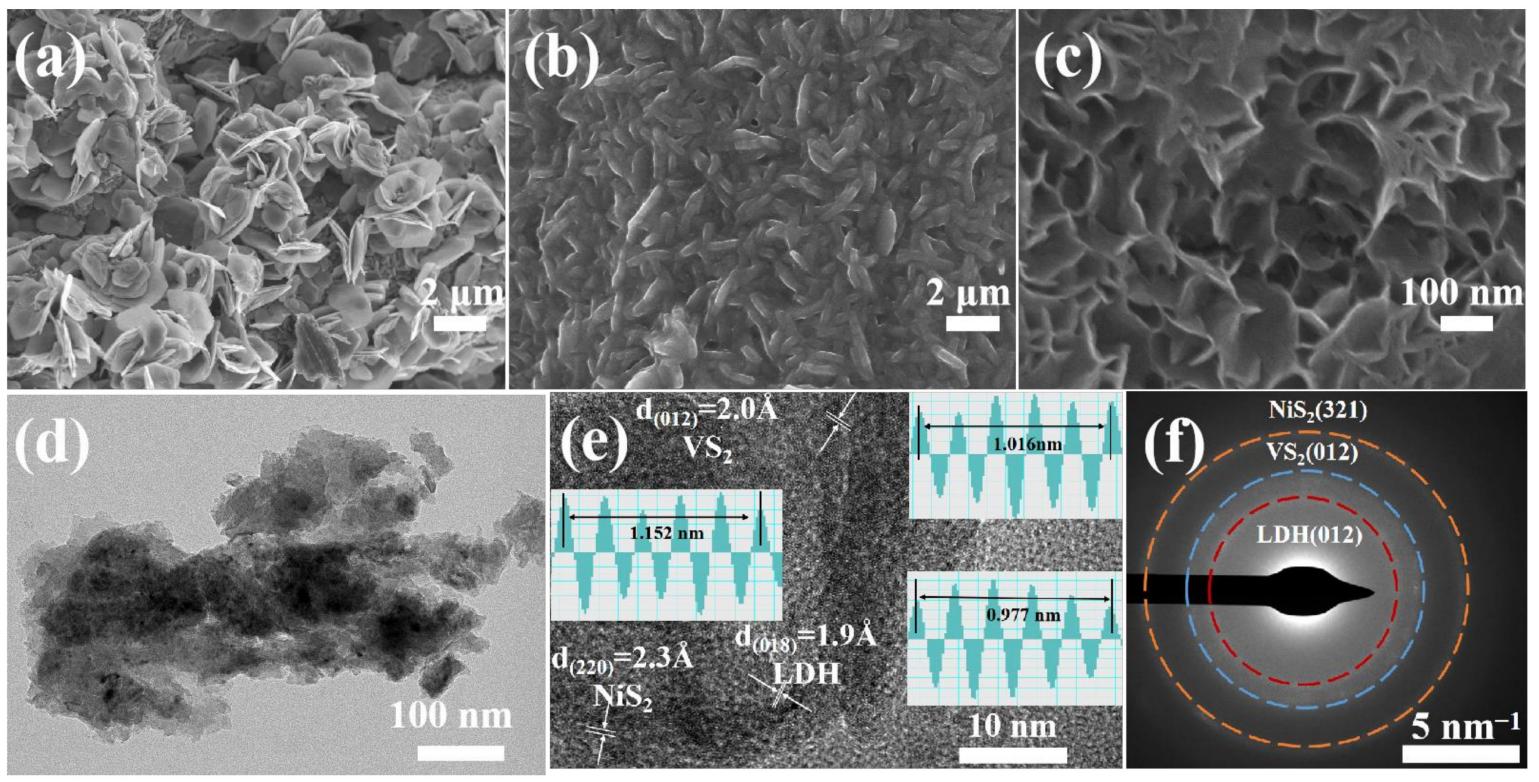
SEM images of (**a**) VS_2_, (**b**) 1:5 NiS_2_/VS_2,_ and (**c**) 350 s NiFe LDH/NiS_2_/VS_2_. (**d**) TEM, (**e**) HRTEM image and (**f**) SAED pattern of 350 s NiFe LDH/NiS_2_/VS_2_.

**Figure 2:**
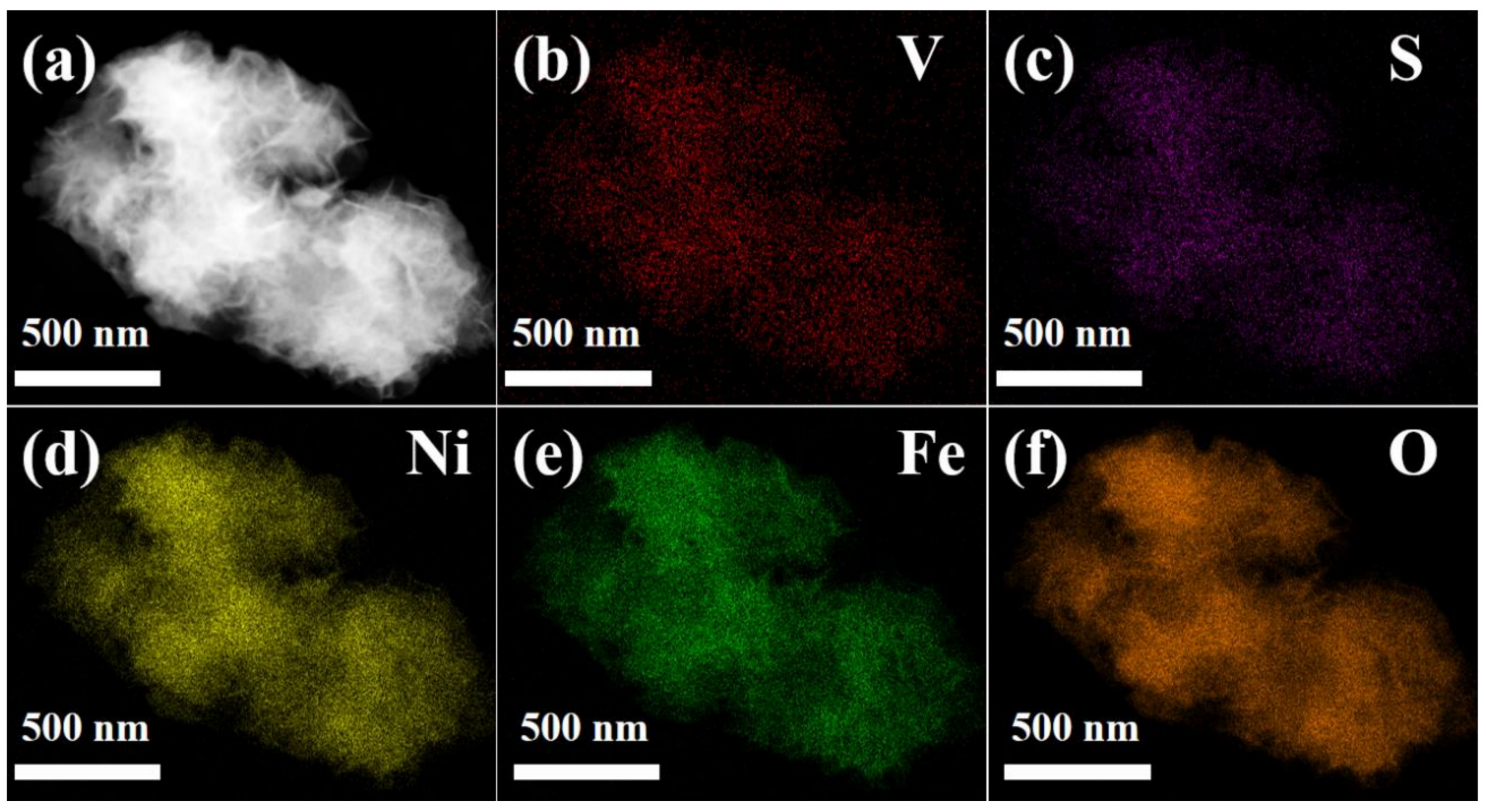
(**a**) High-angle annular dark-field scanning transmission electron microscopy (HAADF-STEM) image of NiFe LDH/NiS_2_/VS_2_ and the corresponding elemental mapping of (**b**) V, (**c**) S, (**d**) Ni, (**e**) Fe and (**f**) O in NiFe LDH/NiS_2_/VS_2_.

The morphology of the electrocatalysts was studied using SEM and TEM. As shown in Figure 1a, the pure VS_2_ exhibits a nanoflower-like structure, which is distinguished from the NiS_2_/VS_2_ by a nanosheet morphology (Figure 1b). By contrast, the NiFe LDH/NiS_2_/VS_2_ has a thin nanosheets structure (Figure 1c), which was beneficial for exposing more active sites. Furthermore, the TEM image (Figure 1d) reveals that NiFe LDH/NiS_2_/VS_2_ exhibits a nanosheet structure. An interplanar spacing of 2.0, 2.3 and 1.9 Å is shown in the HRTEM image (Figure 1e) and can be identified as the (012) plane of VS_2_, (220) plane of NiS_2_ and (018) plane of NiFe LDH, respectively [44,45,46]. As illustrated in Figure 1f, the selected area electron diffraction (SAED) reveals that the (012) plane of NiFe LDH, (012) plane of VS_2_ and (321) plane of NiS_2_ appeared in the NiFe LDH/NiS_2_/VS_2_ sample, suggesting the successful construction of the NiFe LDH/NiS_2_/VS_2_ electrode [44,47,48]. The EDS-mapping measurement (Figure 2) was employed to investigate the element distribution of NiFe LDH/NiS_2_/VS_2_, revealing an evenly distribution of V, S, Ni, Fe and O elements in the NiFe LDH/NiS_2_/VS_2_.

**Figure 3:**
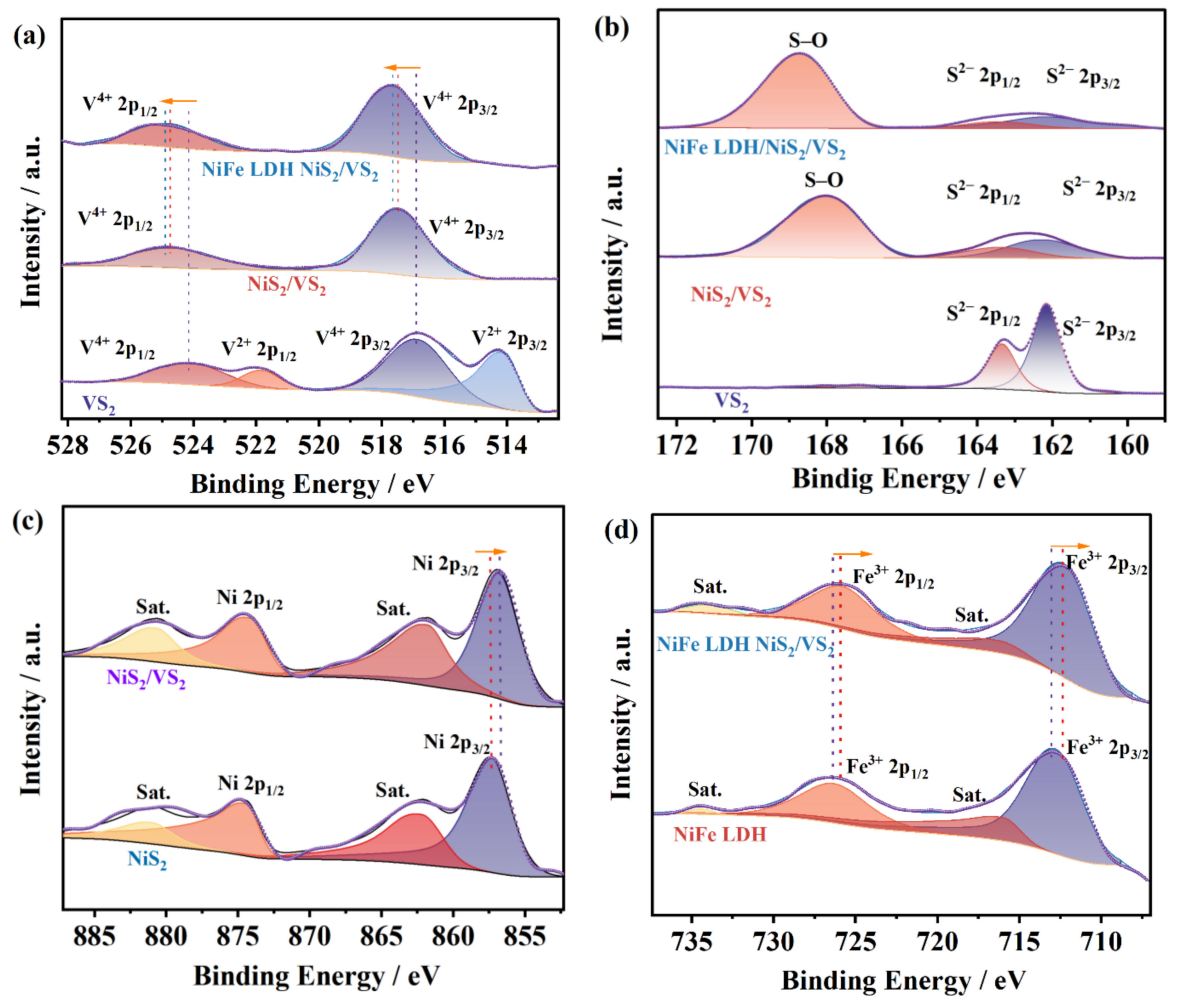
(**a**) High-resolution XPS spectra of (**a**) V 2p, (**b**) S 2p, (**c**) Ni 2p and (**d**) Fe 2p of the prepared VS_2_, NiS_2_/VS_2_ and NiFe LDH/NiS_2_/VS_2_.

To investigate the superficial element composition and the chemical state of the respective elements of the as-prepared electrocatalysts, X-ray photoelectron spectroscopy (XPS) characterization was utilized for the VS_2_, NiS_2_/VS_2_ and NiFe LDH/NiS_2_/VS_2_. Compared with the XPS survey spectra of the VS_2_ (Figure S1b), an obvious signal of the Ni element appears in the survey spectra of the NiS_2_/VS_2_ and NiFe LDH/NiS_2_/VS_2_ samples. The XPS survey of NiFe LDH/NiS_2_/VS_2_ reveals the co-existence of Ni, Fe, V, O and S elements, which corresponds to the result of the EDS-mapping images. As revealed in Figure 3a, the high-resolution XPS spectrum of V for VS_2_ contains typical characteristic peaks at 514.12 and 521.62 eV, which can be related to the V^2+^ 2p_3/2_ and V^2+^ 2p_1/2_, respectively [49,50]. Additionally, there are two dominant peaks at 516.87 and 524.10 eV that can be indexed to the V^4+^ 2p_3/2_ and V^4+^ 2p_1/2_, respectively [49,50,51]. By contrast, the V^4+^ 2p_3/2_ peak around 517.49 eV and V^4+^ 2p_1/2_ around 524.72 eV of NiS_2_/VS_2_ display a positive shift, demonstrating the electronic interaction between NiS_2_ and VS_2_. Similarly, the V 2p XPS pattern of NiFe LDH/NiS_2_/VS_2_ exhibits the two characteristic peaks at 517.61 and 524.86 eV, which can be ascribed to the V^4+^ 2p_3/2_ and V^4+^ 2p_1/2_, respectively [49,50,51]. Consequently, this indicates that the valence state of V does not change after the electrodeposition of NiFe LDH, while an obvious positive shift occurs for V^4+^ 2p_3/2_ and V^4+^ 2p_1/2_ in NiFe LDH/NiS_2_/VS_2_ compared with NiS_2_/VS_2_ and VS_2_. In the S 2p XPS survey of VS_2_, NiS_2_/VS_2_ and NiFe LDH/NiS_2_/VS_2_ (Figure 3b), the peaks at 162.2 and 163.4 eV can be fitted to the S^2-^ 2p_3/2_ and S^2-^ 2p_1/2_, respectively [50,52]. Moreover, the peak at 168.5 eV in the S 2p XPS of NiFe LDH/NiS_2_/VS_2_ can be attributed to the S-O bond, owing to slight oxidization [38].

As revealed in Figure 3c, the high-resolution Ni 2p XPS survey of NiS_2_/VS_2_ reveals a negative shift compared with NiS_2_, further suggesting the strong interfacial electronic interaction of NiS_2_ and VS_2_. In detail, the Ni 2p XPS of NiS_2_ displays a peak at 857.23 eV with a satellite peak of 862.45 eV and a peak at 874.44 eV with a satellite peak of 881.11 eV, belonging to the Ni 2p_3/2_ and Ni 2p_1/2_ of Ni^2+^, respectively [53,54,55]. In comparison, the characteristic peaks of Ni 2p_3/2_ and Ni 2p_1/2_ of NiS_2_/VS_2_ shift to 856.83 and 874.30 eV, respectively. For further exploring the potential effects of the introduction of NiFe LDH, the high-resolution Fe 2p XPS of NiFe LDH and NiFe LDH/NiS_2_/VS_2_ was measured. As revealed in Figure 3d, the peaks of Fe^3+^ 2p_3/2_ and Fe^3+^ 2p_1/2_ in the NiFe LDH/NiS_2_/VS_2_ are separately located at 712.33 and 725.84 eV, which reveals a negative shift of Fe 2p XPS compared with the peaks of Fe^3+^ 2p_3/2_ (713.10 eV) and Fe^3+^ 2p_1/2_ (726.5eV) in the NiFe LDH [55,56,57].

### 2.2. Electrocatalytic Oxygen Evolution Reaction Performance

**Figure 4:**
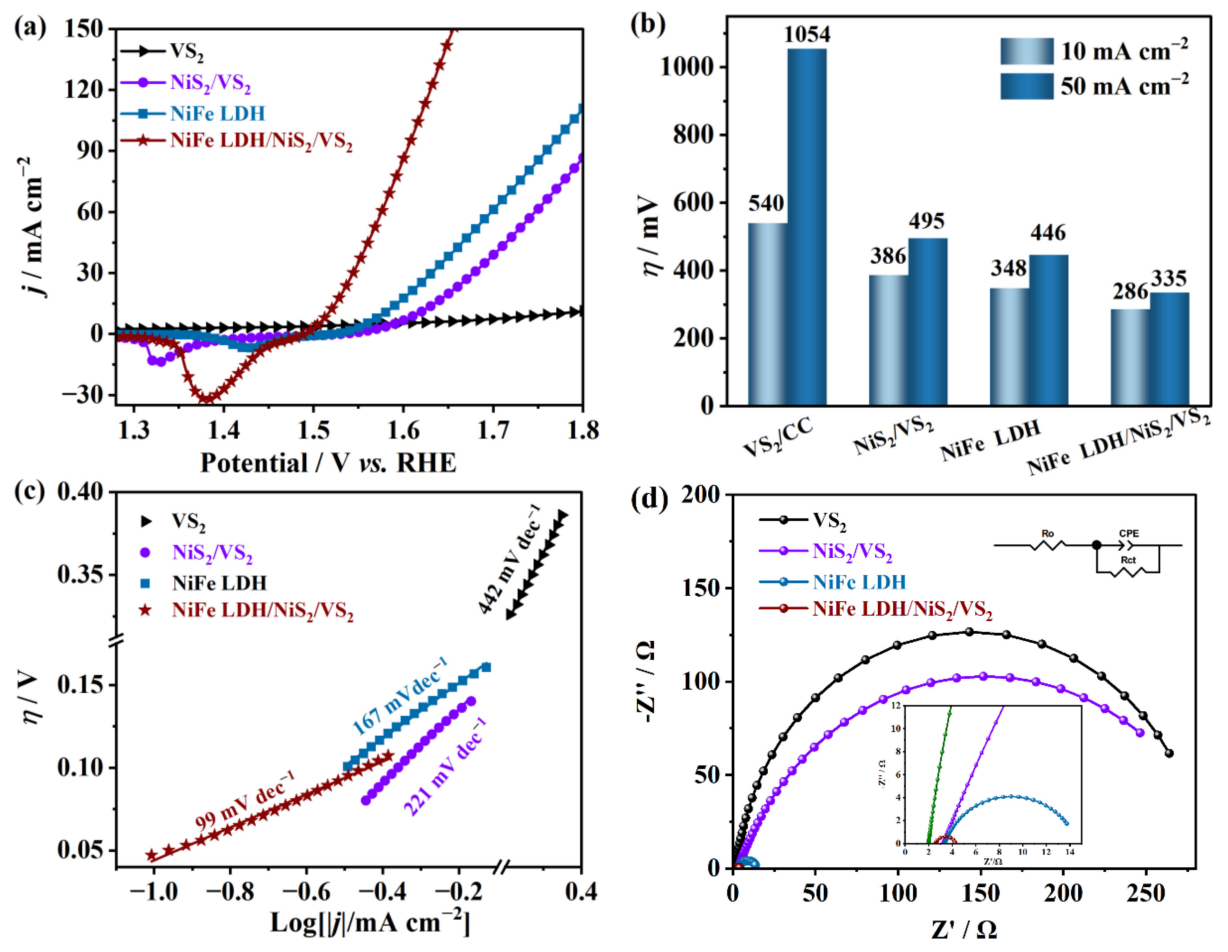
Electrochemical OER performance of the as-prepared VS_2_, NiS_2_/VS_2_, NiFe LDH and NiFe LDH/NiS_2_/VS_2_ samples: (**a**) polarization curves, (**b**) the corresponding overpotential to deliver a current density of 10 and 50 mA cm^−2^ in 1.0 M KOH, (**c**) the Tafel plots and (**d**) the EIS spectra.

The electrocatalytic OER performance of the prepared products (VS_2_, NiS_2_/VS_2_, NiFe LDH and NiFe LDH/NiS_2_/VS_2_) was evaluated in 1.0 M KOH with a typical three-electrode system. As shown in Figure 4a,b, the VS_2_ shows poor electrocatalytic performance for OER, which exhibits a high overpotential of 540 mV to reach a current density of 10 mA cm^−2^, suggesting that the VS_2_ is inactive for electrocatalytic OER. Obviously, the NiS_2_/VS_2_ heterogeneous structure exhibits enhanced electrocatalytic activity for OER, revealing that the formed multi-phase interface is a key factor in boosting the electrocatalytic activity of OER. Figure S2a shows the electrocatalytic OER activity of the VS_2_-based electrocatalysts with different additive ratios of NiSO_4_, in which the 1:5 NiS_2_/VS_2_ (denoted as NiS_2_/VS_2_) exhibits an optimal electrocatalytic activity with an overpotential of 386 mV at 10 mA cm^−2^. Additionally, the electrodeposition time of NiFe LDH was optimized, ranging from 0 to 400 s. Figure S2b reveals that the 350 s NiFe LDH/NiS_2_/VS_2_ exhibits superior electrocatalytic activity when the electrodeposition time of NiFe LDH is 350 s, which has been denoted as NiFe LDH/NiS_2_/VS_2_. The NiFe LDH/NiS_2_/VS_2_ exhibits better electrocatalytic performance for OER compared with VS_2_, NiS_2_/VS_2_ and NiFe LDH, which provides a lower overpotential of 286 mV to deliver a current density of 10 mA cm^−2^. Additionally, the LSV curves of NiFe LDH/NiS_2_/VS_2_ and NiFe LDH show small peaks around 1.35 V, which can be interpreted as the redox reactions of the Ni species. These results imply that the formed heterogeneous structure and NiFe LDH decoration are beneficial to improving the electrocatalytic performance of OER.

Moreover, the electrocatalytic kinetics of as-prepared catalysts were evaluated by measuring Tafel plots. As shown in Figure 4c, the NiFe LDH/NiS_2_/VS_2_ exhibits a smaller Tafel slope of 99 mV dec^−1^ than NiFe LDH (167 mV dec^−1^), NiS_2_/VS_2_ (221 mV dec^−1^) and VS_2_ (442 mV dec^−1^), suggesting the facilitated catalytic kinetics of NiFe LDH/NiS_2_/VS_2_ for OER. The electrochemical impedance spectroscopy (EIS) measurement was utilized to evaluate the charge transfer properties. As Figure 4d depicts, the NiFe LDH/NiS_2_/VS_2_ exhibits the smallest charge transfer resistance (*R_ct_*) among the as-prepared electrocatalysts, indicating that the lower resistance and accelerated electron transfer can be achieved by constructing a heterogeneous structure and decorating NiFe LDH.

**Figure 5:**
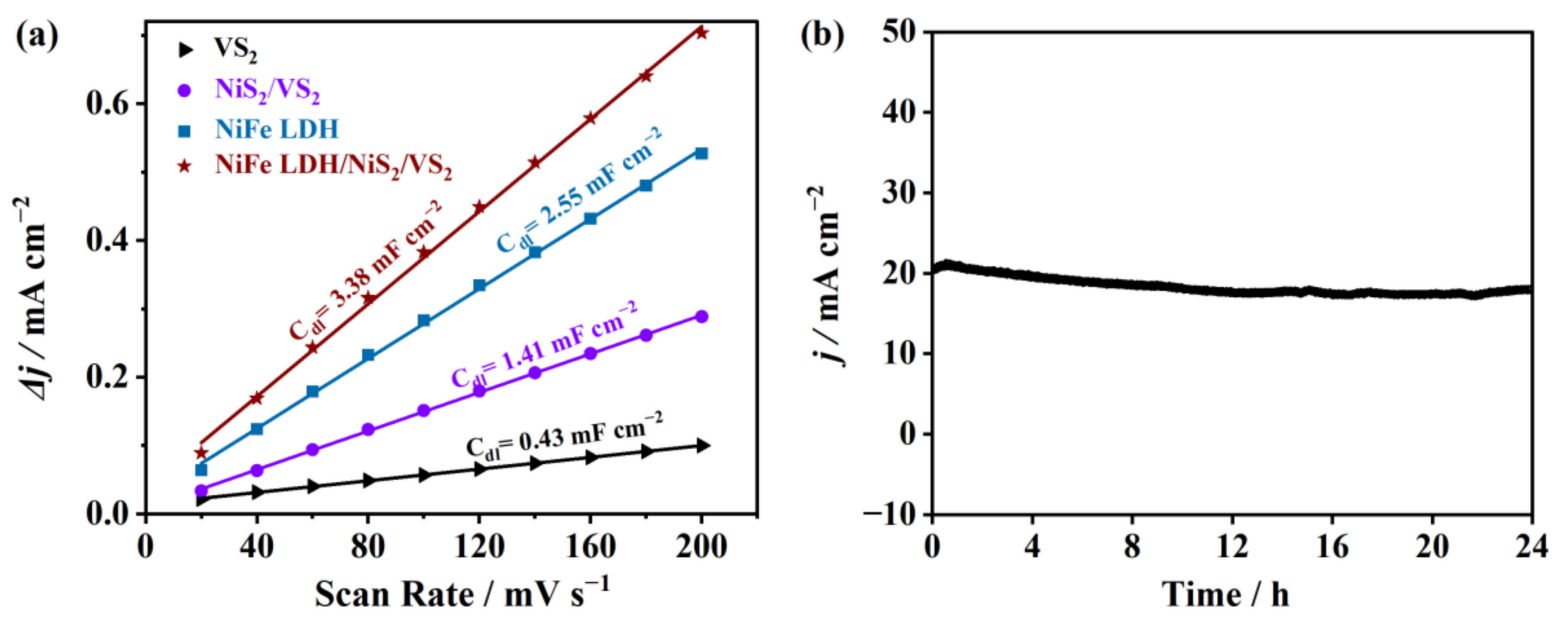
(**a**) The C*_dl_* of the as-prepared VS_2_, NiS_2_/VS_2_, NiFe LDH and NiFe LDH/NiS_2_/VS_2_ samples and (**b**) the long-term stability of NiFe LDH/NiS_2_/VS_2_ under the potential of 0.5 V (vs. SCE).

The electrochemically active surface area (ECSA) of the as-prepared electrocatalysts was evaluated by calculating the C*_dl_* according to the CV curves at various scan rates. As displayed in Figure 5a, the NiFe LDH/NiS_2_/VS_2_ exhibits a much larger C*_dl_* of 3.38 mF cm^−2^ than NiFe LDH (2.55 mF cm^−2^), NiS_2_/VS_2_ (1.41 mF cm^−2^) and VS_2_ (0.43 mF cm^−2^), which can be attributed to the formed heterogeneous structure and the NiFe LDH decoration. Accordingly, the NiFe LDH/NiS_2_/VS_2_ exhibits an ECSA of 84.80 cm^2^, which is higher than that of NiFe LDH (63.75 cm^2^), NiS_2_/VS_2_ (35.25 cm^2^) and VS_2_ (10.75 cm^2^) (Figure S5a). The current density was normalized by the ECSA to evaluate the specific activity. As observed in Figure S5b and Figure 5c, the NiFe LDH/NiS_2_/VS_2_ exhibits intrinsic activity compared to the others, suggesting that the formed heterogeneous structure and modulated electronic structure are favorable to enhancing electrocatalytic activity. Additionally, the electrocatalytic stability of NiFe LDH/NiS_2_/VS_2_ was evaluated by i–t curves in 1.0 M KOH. In Figure 5b, we can observe that the NiFe LDH/NiS_2_/VS_2_ can maintain its superior electrocatalytic activity over 24 h, demonstrating that the prepared NiFe LDH/NiS_2_/VS_2_ can sustain long-term electrolysis without obvious attenuation. Furthermore, an XPS measurement of the NiFe LDH/NiS_2_/VS_2_ was employed after the stability test to investigate the structural stability. As observed in Figure S6, the NiFe LDH/NiS_2_/VS_2_ can maintain its initial chemical composition and chemical state after a long-term stability test. The thin nanosheet structure also can be observed (Figure S7) after long-term stability, demonstrating the structural stability of NiFe LDH/NiS_2_/VS_2_.

### 2.3. Electrocatalytic Hydrogen Evolution Reaction and Overall Water-Splitting Performance

**Figure 6:**
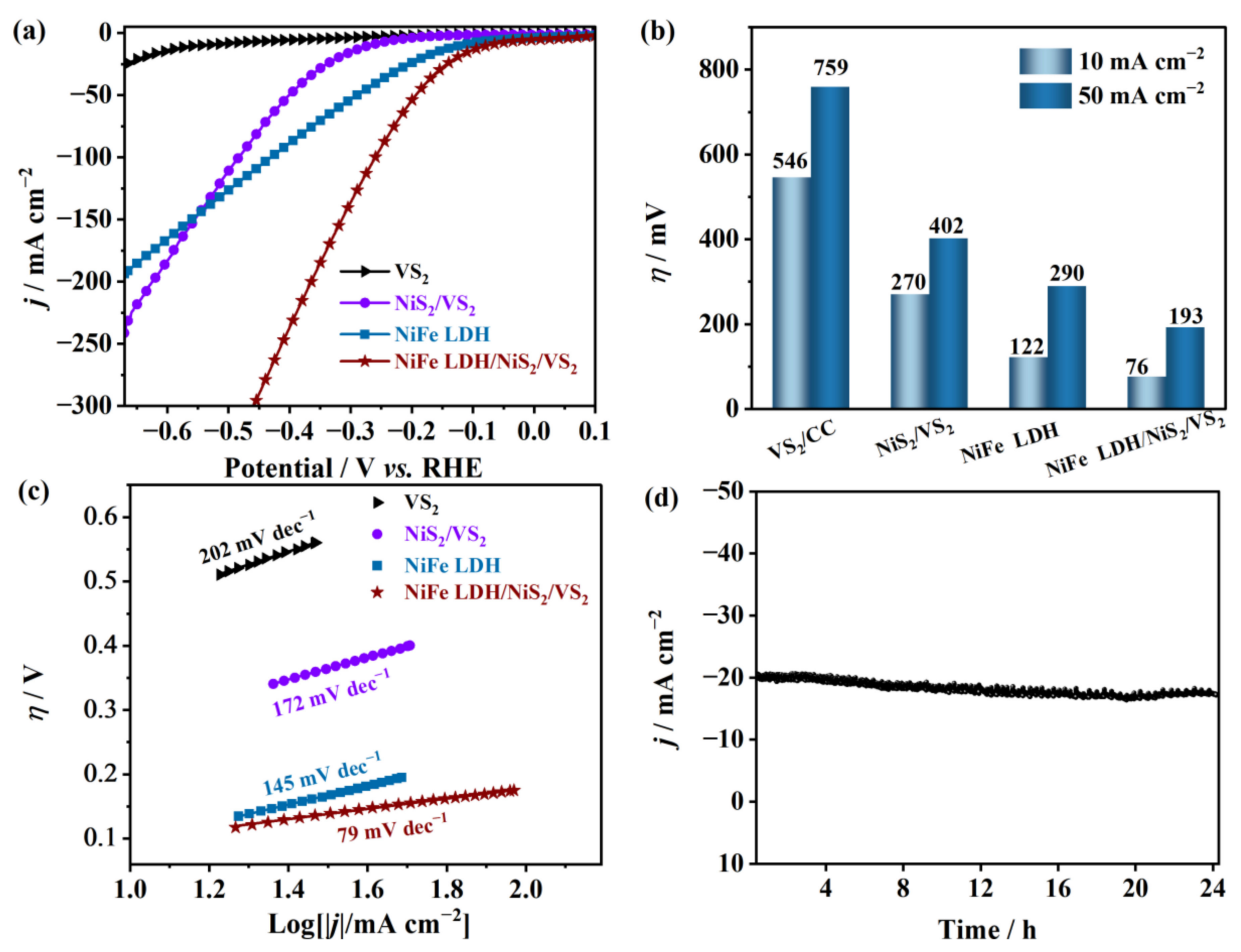
Electrochemical HER performance of the as-prepared VS_2_, NiS_2_/VS_2_, NiFe LDH and NiFe LDH/NiS_2_/VS_2_ samples: (**a**) polarization curves, (**b**) the corresponding overpotential to deliver a current density of 10 and 50 mA cm^−2^ in 1.0 M KOH, (**c**) the Tafel plots and (**d**) the long-term stability text of NiFe LDH/NiS_2_/VS_2_ under the potential of −1.2 V (vs. SCE).

To investigate its potential for efficient overall water splitting, the electrocatalytic performance of the NiFe LDH/NiS_2_/VS_2_ for HER was evaluated in 1.0 M KOH. Figure 6a,b shows that the NiFe LDH/NiS_2_/VS_2_ electrocatalyst exhibits excellent electrocatalytic activity, acquiring a smaller overpotential of 76 mV to deliver a current density of 10 mA cm^−2^, which is greatly superior to that of the VS_2_ (546 mV), NiS_2_/VS_2_ (270 mV) and NiFe LDH (122 mV). The corresponding Tafel plots are illustrated in Figure 6c. The NiFe LDH/NiS_2_/VS_2_ has a smaller Tafel slope of 79 mV dec^−1^ than VS_2_ (202 mV dec^−1^), NiS_2_/VS_2_ (175 mV dec^−1^) and NiFe LDH (145 mV dec^−1^). Moreover, the NiFe LDH/NiS_2_/VS_2_ exhibits remarkable stability during 24 h electrochemical measurement and maintains its electrocatalytic activity for HER (Figure 6d).

**Figure 7:**
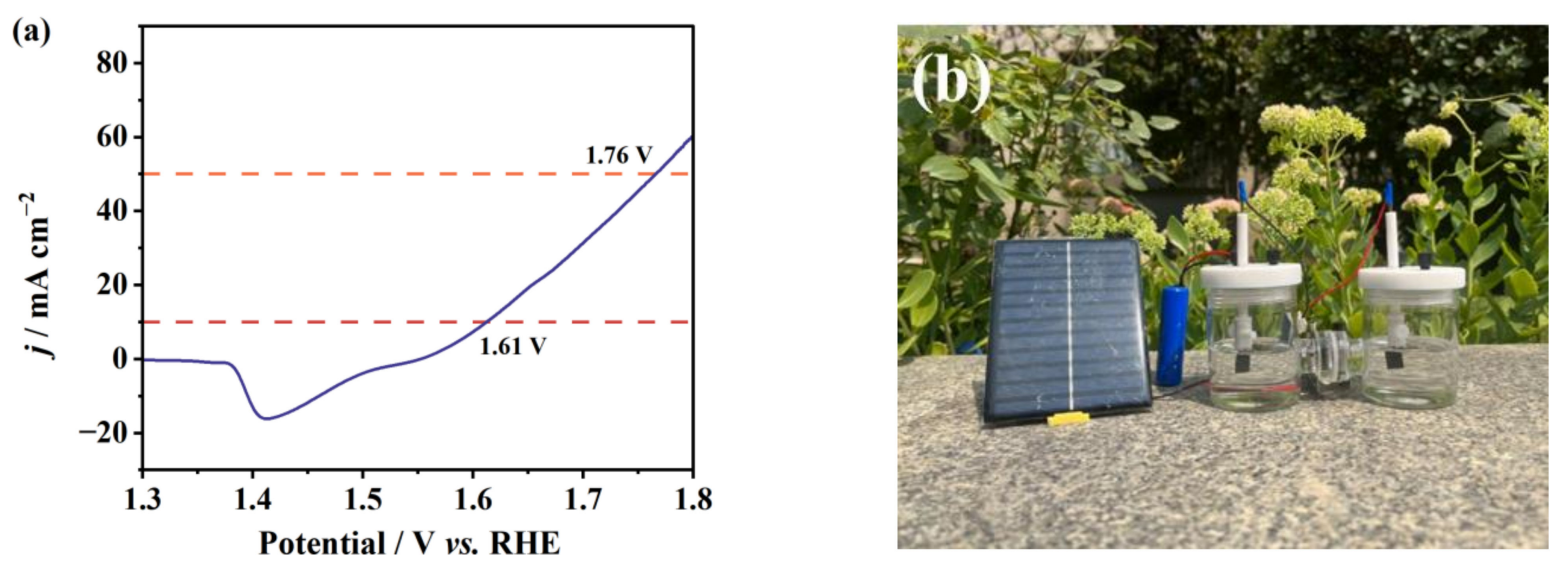
(**a**) The LSV curves of LDH/NiS_2_/VS_2_ (+, −) device for overall water splitting, (**b**) image of the solar-driven water-splitting system in 1.0 M KOH electrolyte.

Electrocatalytic overall water splitting was measured by a two-electrode system composed of NiFe LDH/NiS_2_/VS_2_ in 1.0 M KOH. As shown in Figure 7a, the constructed overall water-splitting system is able to deliver a current density of 10 mA cm^−2^ at 1.61 V, confirming its potential to drive the overall water splitting. Furthermore, a commercial solar cell under 2.0 V was employed to explore its solar-drive overall water-splitting performance. As shown in Figure 7b, a number of microbubbles of H_2_ and O_2_ gas at the cathode and anode, respectively, evolved, which clearly demonstrates the superior electrocatalytic activity of the NiFe LDH/NiS_2_/VS_2_. Furthermore, the corresponding Video S1 recorded this dynamic state.

## 3. Experimental Section

### 3.1. Materials

Ammonium metavanadate (NH_4_VO_3_, AR, Tianjin Damao Chemical Reagent Factory, Tianjin, China), thioacetamide (CH_3_CSNH_2_, Aladdin Industrial Corporation, Shanghai, China), nickel sulfate hexahydrate (NiSO_4_·6H_2_O, AR, Zhongxin Fine Chemical Co., Ltd., Jinan, China), (FeSO_4_·7H_2_O, AR, Sinopharm Chemical Reagent Co., Ltd., Shanghai, China) ammonium hydroxide (NH_4_OH, AR, Tianjin Fuyu Fine Chemical Co., Ltd., Tianjin, China), and carbon cloth (CC, SCI Materials Hub, Taiwan, China) were employed as substrates for the in-situ formation of VS_2_ and NiS_2_/VS_2_ on its surface via hydrothermal synthesis. Potassium hydroxide (KOH, Tianjin Kemiou Chemical Reagent Co., Ltd., Tianjin, China), nitric acid (HNO_3_, AR, Chengdu Kelong Chemical, Chengdu, China), and ethyl alcohol (C_2_H_6_O, AR, Tianjin Fuyu Fine Chemical Co., Ltd., Tianjin, China) were obtained to pretreat CC. Deionized water was prepared in our laboratory. All chemicals mentioned were utilized as received without further purification.

### 3.2. Pretreatment of Carbon Cloth (CC) Substrate

Carbon cloth (CC) with a geometric area of 2 × 4 cm^2^ was sonicated in an ultrasonic bath with deionized water and ethanol (C_2_H_5_OH) severally for 15 min to clean its surface thoroughly. Then, the hydrophilic surface was obtained via pretreatment in concentrated HNO_3_ at 100 °C for 2 h. Finally, the CC surface was fully rinsed with deionized water to remove residual HNO_3_, and the pretreated CC was obtained.

### 3.3. Preparation of 1:5 NiS_2_/VS_2_

A typical procedure to synthesize 1:5 NiS_2_/VS_2_ was performed as follows: 1 mmol NH_4_VO_3_ was added to a weak alkaline solution produced by 25 mL deionized water and 2 mL NH_4_OH while stirring constantly. At the same time, 10 mmol CH_3_CSNH_2_ and 0.2 mmol NiSO_4_·6H_2_O were dispersed in 10 mL deionized water with stirring. The above two solutions were blended and stirred vigorously at room temperature for 1 h. The mixture solution and prepared CC (2 × 4 cm^2^) were sealed and maintained at 160 °C for 24 h in a 50 mL Teflon-lined autoclave. The sample was cooled to an ambient temperature naturally after the reaction. Then, the prepared sample was rinsed several times with absolute ethanol and deionized water, respectively. Finally, it was dried at 60 °C for 6 h, and the 1:5 NiS_2_/VS_2_ was obtained.

For investigating the effect of NiS_2_ on electrocatalytic HER and OER activities, VS_2_, 1:10 NiS_2_/VS_2_, 1:7.5 NiS_2_/VS_2,_ and 1:2 NiS_2_/VS_2_ were synthesized by a similar method with 1:5 NiS_2_/VS_2_ in terms of finely varying the additive amount of NiSO_4_·6H_2_O. More specifically, 0 mmol, 0.1 mmol, 0.133 mmol, and 0.5 mmol NiSO_4_·6H_2_O were added into the precursor solution for preparing VS_2_, 1:10 NiS_2_/VS_2_, 1:7.5 NiS_2_/VS_2,_ and 1:2 NiS_2_/VS_2_, individually.

### 3.4. Preparation of 350 s NiFe LDH/NiS_2_/VS_2_

The hydrothermal synthetic method for 1:5 NiS_2_/VS is based on the above-mentioned method. NiFe LDH was grown through an electrodeposition method on the surface of 1:5 NiS_2_/VS_2_. Then, 0.025 M NiSO_4_ and 0.0125 M FeSO_4_ solutions were homogeneously mixed in a three-electrode system with saturated calomel electrode (SCE), NiS_2_/VS_2_, and Pt plate as the reference, working, and counter electrode, respectively. The electrodeposition process was operated at a constant voltage of -1.0 V (vs. SCE) at room temperature with different electrodeposition times (250 s, 300 s, 350 s and 400 s), and the corresponding samples were denoted 250 s NiFe LDH/NiS_2_/VS_2_, 300 s NiFe LDH/NiS_2_/VS_2_, 350 s NiFe LDH/NiS_2_/VS_2_ and 400 s NiFe LDH/NiS_2_/VS_2_, respectively. Finally, these as-obtained catalytic electrodes were gently washed several times using deionized water and then dried naturally.

As a reference, NiFe LDH was grown on pretreated CC using the same electrodeposition synthetic process for 350 s with the absence of 1:5 NiS_2_/VS_2_.

### 3.5. Structural Characterization

The phase composition was determined on an XRD-7000 s device (Shimadzu, Kyoto, Japan) with Cu Kα radiation (λ = 1.5418 Å) in a 2 Theta scanning range from 10 to 80° at a current of 40 mA and a voltage of 40 kV. The morphology of the electrocatalysts was measured by means of a scanning electron microscope (SEM, Sigma 300, ZEISS, Oberkochen, Germany), and the energy dispersive spectrum (EDS) was recorded on Oxford Xplore. The transmission electron microscopy (TEM) and high-resolution TEM (HRTEM) images were determined using a TEM instrument (Talos F200×, FEI, Hillsboro, OR, USA) with a field-emission gun operated at 200 kV, and the EDS was recorded. Surface chemical analysis was performed by X-ray photoelectron spectroscopy (XPS, Thermo Scientific, K-Alpha Nexsa, Waltham, MA, USA) using monochromatic Al Kα radiation (1486.6 eV).

### 3.6. Electrochemical Measurements

The electrocatalytic performances for both HER and OER were carried out on a CHI660E electrochemical workstation using a traditional three-electrode system and an H-type electrochemical cell in an electrolyte of 1.0 M KOH. The as-prepared electrode with a geometric area of 1.0 cm^2^, the saturated calomel electrode (SCE), and the Pt plate were individually employed as working, reference and counter electrodes. The working electrode and SCE reference electrode were placed in the same chamber, and the Pt plate was placed in the others. All potentials in the present work were measured compared to reversible hydrogen evolution (RHE) in accordance with the following formula (E_RHE_ = E_SCE_ + 0.0591pH + E^θ^_SCE_) [58,59]. Before testing, the working electrode was stabilized and the surface was cleaned using a cyclic voltammetry (CV) method. The catalytic activity of each electrocatalyst for HER and OER was estimated through linear sweep voltammetry (LSV) at a scanning rate of 5 mV s^−1^. According to the formula (ECSA = C*_dl_*/C*_s_*), the electrochemical active area (ECSA) was determined by calculating the double-layer capacitance (C*_dl_*) values, where C*_dl_* can be calculated by CV measurements in the non-Faradaic region with variable scan rates, and C*_s_* represents the specific capacitance [60,61]. The Tafel plots were measured in the same testing system for unraveling the electrocatalytic kinetics of the as-prepared samples. To probe the electron transfer, the electrochemical impedance spectroscopy (EIS) was measured by application of an alternating voltage of 10 mV amplitude with a frequency ranging from 0.1 Hz to 100 kHz. The electrocatalytic stability was evaluated by current–time curves (i–t). Moreover, the potentials related to OER and HER were set as 0.5 V (vs. SCE) and −1.2 V (vs. SCE), respectively. The electrocatalytic overall water-splitting activity of the as-prepared electrocatalyst was measured by a two-electrode configuration, in which the NiFe LDH/NiS_2_/VS_2_ was employed as an electrocatalyst for the HER cathode and the OER anode in 1.0 M KOH.

## 4. Conclusions

In conclusion, NiFe LDH/NiS_2_/VS_2_ heterostructured electrocatalysts have been designed as bifunctional high-efficiency electrocatalysts to split water using a simple method, in which VS_2_ with metallic properties and the heterostructured construction endow enhanced electron conductivity and smaller charge transfer resistance to the NiFe LDH/NiS_2_/VS_2_ electrocatalyst. Furthermore, the formation of a nanoflower structure through decorating NiFe LDH on the surface of NiS_2_/VS_2_ via a facile electrodeposited method realizes more exposed active sites. The interfacial electron interaction between NiS_2_/VS_2_ and NiFe LDH was expected to enhance its electrocatalytic activity for overall water splitting. Remarkably, the NiFe LDH/NiS_2_/VS_2_ exhibits superior electrocatalytic activity compared to VS_2_, NiS_2_/VS_2_ and NiFe LDH; it can achieve a current density of 10 mA cm^−2^ at an overpotential of 76 and 286 mV for HER and OER, respectively. At the same time, the corresponding Tafel plots also decreased significantly. Furthermore, the two-electrode system for overall water splitting was constructed by employing the NiFe LDH/NiS_2_/VS_2_ as a bifunctional electrocatalyst, which requires an overpotential of 380 mV to achieve a current density of 10 mA cm^−2^. This study presents a tactic base regarding the interfacial regulation of NiFe LDH, NiS_2_ and VS_2_ to enhance electrocatalytic OER and HER activity, which is helpful for developing highly efficient electrocatalysts to split water.

## Data Availability

The data presented in this study are available on request from the corresponding author.

## Supplementary Materials

The following supporting information can be downloaded at: https://www.mdpi.com/article/10.3390/molecules29050951/s1, Figure S1. (a) XRD patterns and (b) XPS survey spectra of the prepared VS_2_, NiS_2_/VS_2_ and NiFe LDH/NiS_2_/VS_2_. Figure S2. Electrocatalytic OER activity of (a) the VS_2_ and NiS_2_/VS_2_ with different addition ratios of nickel source and (b) the NiFe LDH/NiS_2_/VS_2_ with different electrodeposition times. Figure S3. Cyclic voltammetry curves of the (a) VS_2_, (b) NiS_2_/VS_2_, (c) 250 s NiFe LDH/NiS_2_/VS_2_, (d) 300 s NiFe LDH/NiS_2_/VS_2_, (e) 350 s NiFe LDH/NiS_2_/VS_2_, (f) 400 s NiFe LDH/NiS_2_/VS_2_ and (g) 350 s NiFe LDH in the non-faradaic region with different scan rates. (h) The C*_dl_* value of the corresponding samples. Figure S4. Electrocatalytic HER activity of (a) the VS_2_ and NiS_2_/VS_2_ with different addition ratios of nickel source and (b) the NiFe LDH/NiS_2_/VS_2_ with different electrodeposition times. Figure S5. (a) ECSA and the electrocatalytic activity normalized by ECSA for (b) OER and (c) HER of the as-prepared VS_2_, NiS_2_/VS_2_, NiFe LDH and NiFe LDH/NiS_2_/VS2. Figure S6. High-resolution XPS survey of the NiFe LDH/NiS_2_/VS_2_ before and after stability test; (a) V 2p, (b) S 2p, (c) Ni 2p and (d) Fe 2p. Table S1. Comparisons of HER and OER activity of NiFe LDH/NiS_2_/VS_2_ with other electrocatalysts in 1.0 M KOH. Figure S7. SEM of NiFe LDH/NiS_2_/VS_2_ after stability test. Video S1: The dynamic state of the constructed overall water-splitting system. Refs [62,63,64,65,66,67,68,69,70,71,72,73,74] are cited in the Supplementary Materials.
