# Supplementary material for: Interfacial Interaction in NiFe LDH/NiS2/VS2 for Enhanced Electrocatalytic Water Splitting
Source: Molecules. 2024 Feb 21;29(5):951. doi: 10.3390/molecules29050951 (PMC10934830; doi:10.3390/molecules29050951)
Supplement: Supplementary file 1 [file molecules-29-00951-s001.zip › molecules-2865315-supplementary.pdf]

Supporting Information

Interfacial interaction in NiFe LDH/NiS_2/VS_2 for enhanced electrocatalytic water splitting

Tingxia Wang *, Xu Zhang, Xiaojiao Yu *, Junpeng Li, Zongbin Liu and Jinfen Niu

School of Science, Xi'an University of Technology, Xi’an, Shaanxi, 710054, China

E-mail: wangtingxia@xaut.edu.cn (Tingxia Wang). yxjw@xaut.edu.cn (Xiaojiao Yu)

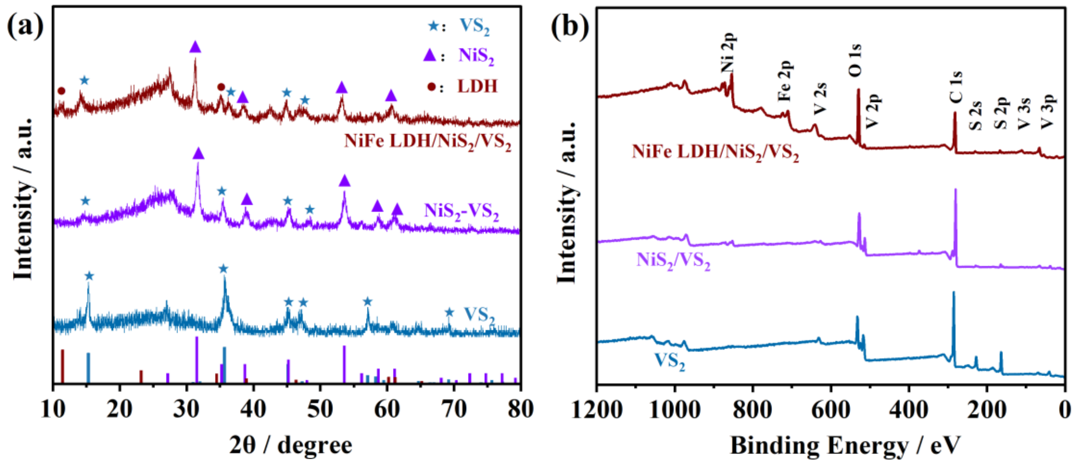

Figure S1. (a)XRD patterns and (b) XPS survey spectra of the prepared VS_2, NiS_2/VS_2 and NiFe LDH/NiS_2/VS_2.

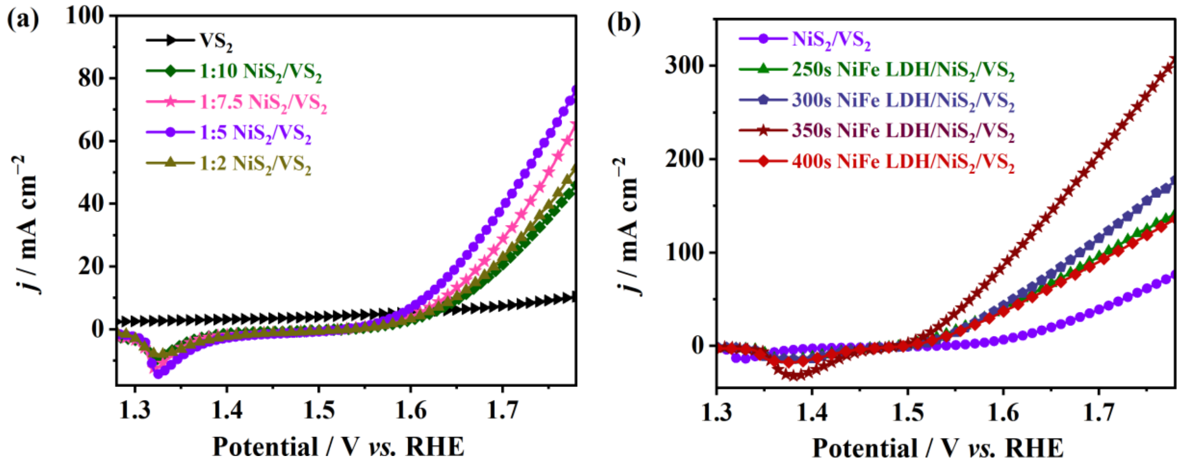

Figure S2. Electrocatalytic OER activity of (a) the VS_2 and NiS_2/VS_2 with different

addition ratio of nickel source and (b) the NiFe LDH/NiS_2/VS_2 with different electrodeposition time.

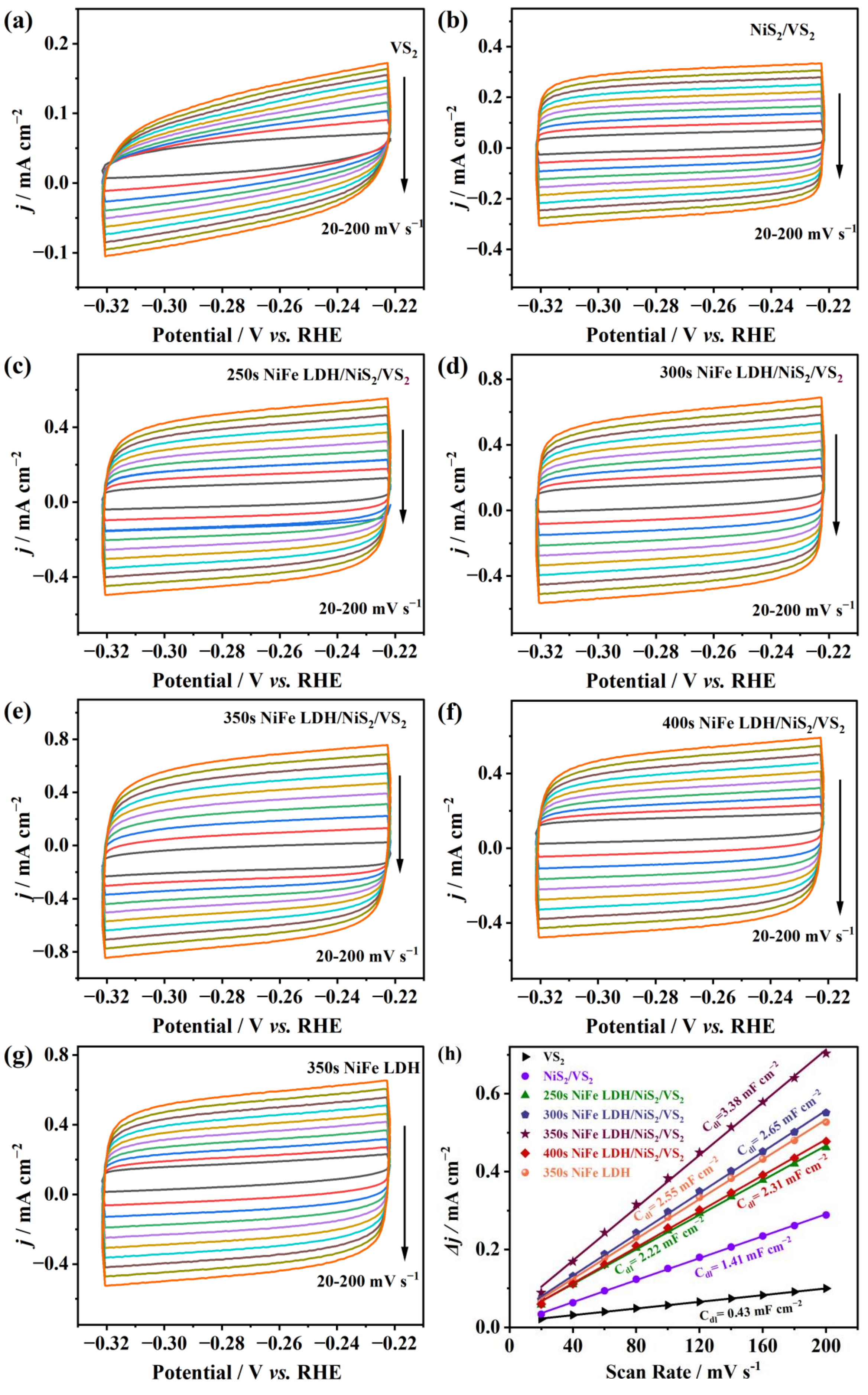

(a)
VS$_2$
20-200 mV s^{-1}
j / mA cm^{-2}
Potential / V vs. RHE
(b)
NiS$_2$/VS$_2$
20-200 mV s^{-1}
j / mA cm^{-2}
Potential / V vs. RHE
(c)
250s NiFe LDH/NiS$_2$/VS$_2$
20-200 mV s^{-1}
j / mA cm^{-2}
Potential / V vs. RHE
(d)
300s NiFe LDH/NiS$_2$/VS$_2$
20-200 mV s^{-1}
j / mA cm^{-2}
Potential / V vs. RHE
(e)
350s NiFe LDH/NiS$_2$/VS$_2$
20-200 mV s^{-1}
j / mA cm^{-2}
Potential / V vs. RHE
(f)
400s NiFe LDH/NiS$_2$/VS$_2$
20-200 mV s^{-1}
j / mA cm^{-2}
Potential / V vs. RHE
(g)
350s NiFe LDH
20-200 mV s^{-1}
j / mA cm^{-2}
Potential / V vs. RHE
(h)
VS$_2$
NiS$_2$/VS$_2$
250s NiFe LDH/NiS$_2$/VS$_2$
300s NiFe LDH/NiS$_2$/VS$_2$
350s NiFe LDH/NiS$_2$/VS$_2$
400s NiFe LDH/NiS$_2$/VS$_2$
350s NiFe LDH
C$_{dl}$=3.38 mF cm^{-2}
C$_{dl}$= 2.65 mF cm^{-2}
C$_{dl}$= 2.55 mF cm^{-2}
C$_{dl}$= 2.31 mF cm^{-2}
C$_{dl}$= 2.22 mF cm^{-2}
C$_{dl}$= 1.41 mF cm^{-2}
C$_{dl}$= 0.43 mF cm^{-2}
Δj / mA cm^{-2}
Scan Rate / mV s^{-1}

Figure S3. Cyclic voltammetry curves of the (a) VS_2, (b) NiS_2/VS_2, (c) 250s NiFe LDH/NiS_2/VS_2, (d) 300s NiFe LDH/NiS_2/VS_2, (e) 350s NiFe LDH/NiS_2/VS_2, (f) 400s NiFe LDH/NiS_2/VS_2 and (g) 350s NiFe LDH at non-faradaic region with a different scan rates. (h) The C_{dl} value of the corresponding samples.

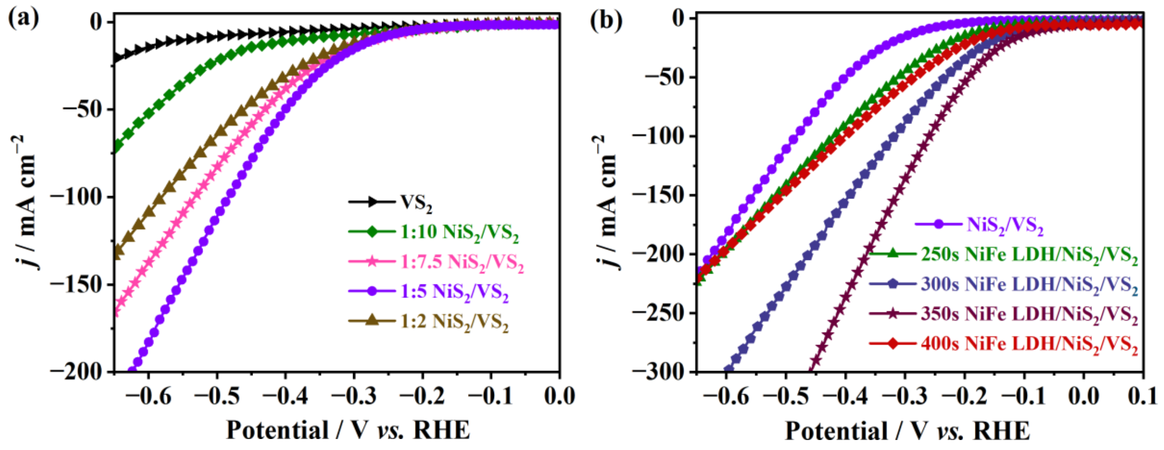

Figure S4. Electrocatalytic HER activity of (a) the VS_2 and NiS_2/VS_2 with different addition ratio of nickel source and (b) the NiFe LDH/NiS_2/VS_2 with different electrodeposition time.

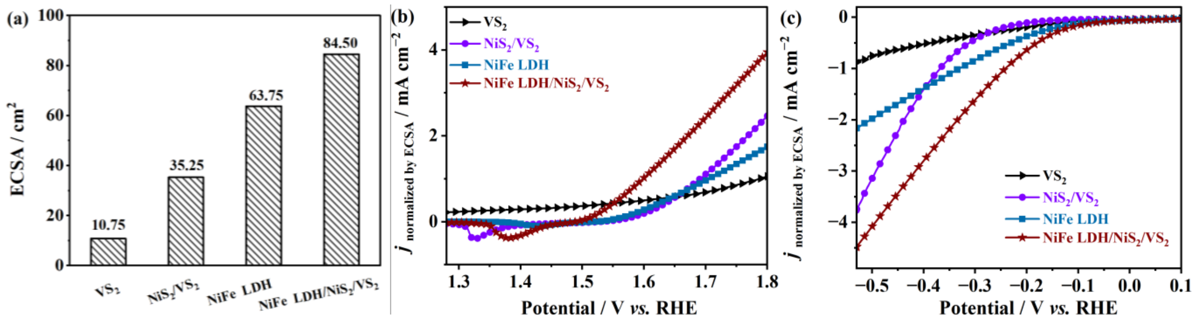

Figure S5. (a) ECSA and the elcctrocatalytic activity normalized by ECSA for (b) OER and (c) HER of the as-prepared VS_2, NiS_2/VS_2, NiFe LDH and NiFe LDH/NiS_2/VS_2.

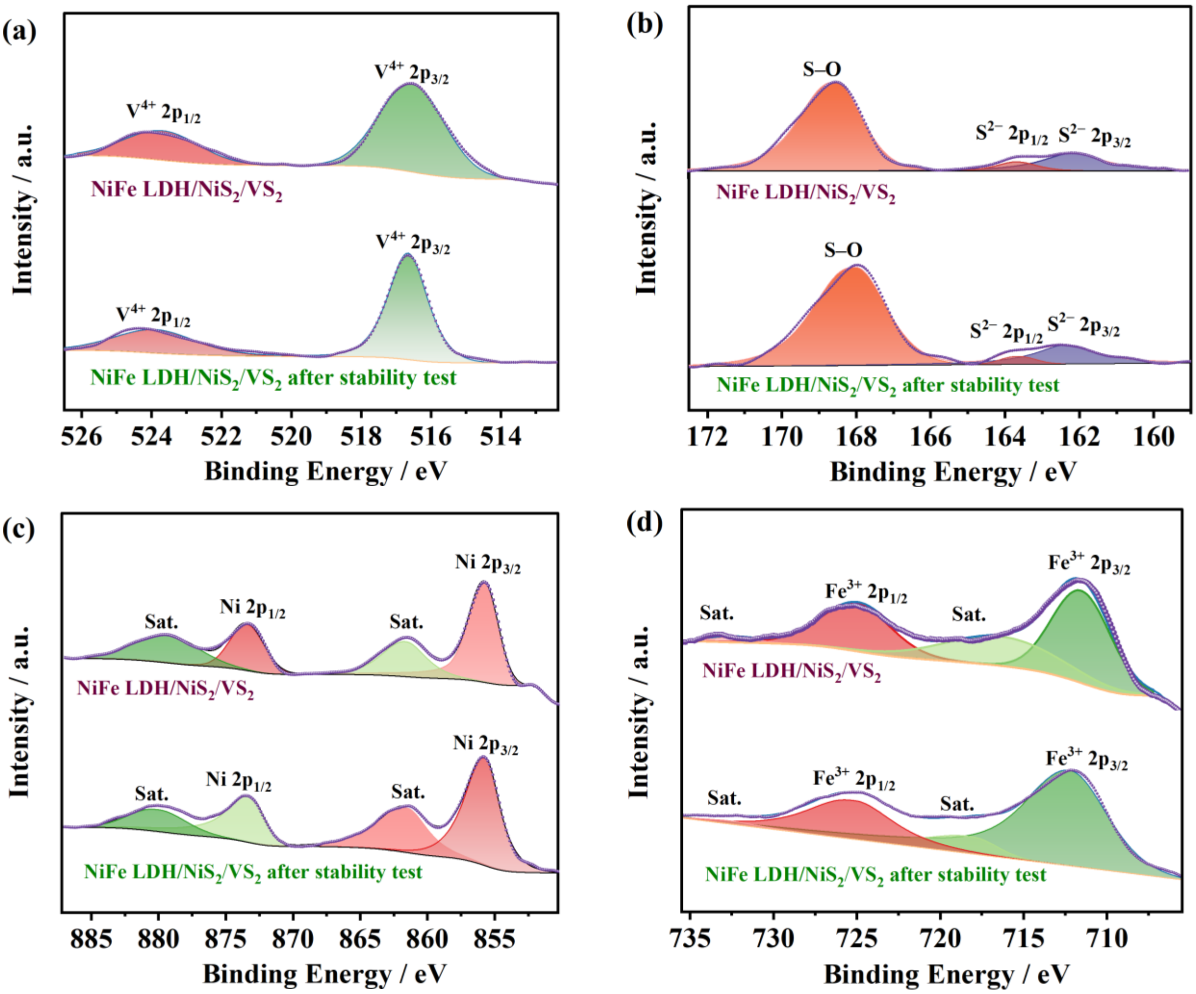

Figure S6. High-resolution XPS survey of the NiFe LDH/NiS_2/VS_2 before and after stability test; (a) V 2p, (b) S 2p, (c) Ni 2p and (d) Fe 2p.

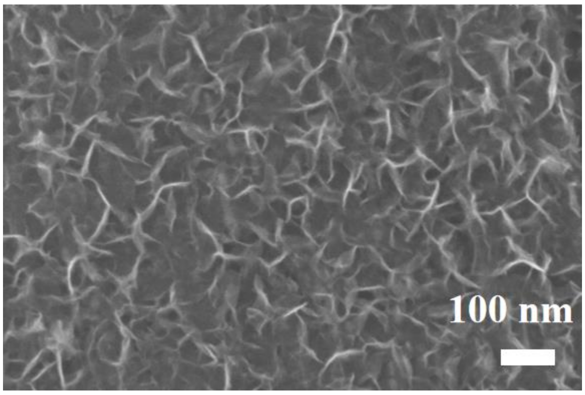

Figure S7. SEM of NiFe LDH/NiS_2/VS_2 after stability test.

Table S1. Comparisons of HER and OER activity of NiFe LDH/NiS_2/VS_2 with other electrocatalysts.

Catalyst	Electrolyte	HER		OER		Overall voltage @10 mA cm^{-2} (V)	iR compensation	Ref.
		h_{10} (mV)	Tafel slope (mV dec^{-1})	h_{10} (mV)	Tafel slope (mV dec^{-1})			
**NiFe LDH/NiS_2/VS_2**	**1.0 M KOH**	**76**	**79**	**286**	**99**	**1.61**	**no**	**This Work**
VS_2/MoS_2	1.0 M KOH	148	69	-	-	-	yes	[62]
VS_2	1.0 M KOH	197	139	-	-	-	yes	[63]
VS_2/NF	1.0 M KOH	197	134.39	330	87.25	-	yes	[64]
$CoMnS_2$@1T-Fe-VS_2@NF	1.0 M KOH	89	61	260 (20 mA cm^{-2})	51	1.51	yes	[65]
$NiCo_2S_4$@C_3N_4@VS_2	1.0 M KOH	110	71.8	-	-	-	-	[66]
VS_2/rGO	1.0 M KOH	-	-	310	72	-	yes	[67]
V-doped pyrite NiS_2	1.0 M KOH	85	133	-	-	-	yes	[58]
{0 0 1}-NiS_2:Fe	1.0 M KOH	-	-	277	57	-	yes	[68]
Fe-NiS_2@g-C_3N_4	1.0 M KOH	-	-	280	97.3	-	yes	[69]
MoS_2/NiFe LDH	1.0 M KOH	98	95	257	59	1.61	yes	[47]
Fe_3O_4/NiFe LDH/Fe_3O_4	1.0 M KOH	134	141.5	260 (50 mA cm^{-2})	89.7	1.648	yes	[70]
CoNiN@NiFe LDH	1.0 M KOH	150	169	227	58.1	1.63	yes	[71]

NiFe-LDH@Mo-NiS-NiS_2/NF	1.0 M KOH	120	105	261 (50 mA cm^{-2})	86	1.63	yes	[72]
Ni_3S_2/VG@NiCo LDHs	1.0 M KOH	120	87	350 (100 mA cm^{-2})	65	1.66	no	[73]
CoS@NiFe LDH/NF	1.0 M KOH	95	90	250	49	1.65	yes	[74]
